# Influence of Fibroblasts on Mammary Gland Development, Breast Cancer Microenvironment Remodeling, and Cancer Cell Dissemination

**DOI:** 10.3390/cancers12061697

**Published:** 2020-06-26

**Authors:** Angelica Avagliano, Giuseppe Fiume, Maria Rosaria Ruocco, Nunzia Martucci, Eleonora Vecchio, Luigi Insabato, Daniela Russo, Antonello Accurso, Stefania Masone, Stefania Montagnani, Alessandro Arcucci

**Affiliations:** 1 Department of Public Health, University of Naples Federico II, 80131 Naples, Italy; nu.martucci@studenti.unina.it (N.M.); montagna@unina.it (S.M.); 2 Department of Experimental and Clinical Medicine, University “Magna Graecia” of Catanzaro, 88100 Catanzaro, Italy; fiume@unicz.it (G.F.); eleonoravecchio@unicz.it (E.V.); 3 Department of Molecular Medicine and Medical Biotechnology, University of Naples Federico II, 80131 Naples, Italy; mariarosaria.ruocco2@unina.it; 4 Anatomic Pathology Unit, Department of Advanced Biomedical Sciences, School of Medicine, University of Naples Federico II, 80131 Naples, Italy; insabato@unina.it (L.I.); daniela.russo@unina.it (D.R.); 5 Department of General, Oncological, Bariatric and Endocrine-Metabolic Surgery, University of Naples Federico II, 80131 Naples, Italy; antonello.accurso@unina.it; 6 Department of Clinical Medicine and Surgery, University of Naples Federico II, 80131 Naples, Italy; stefania.masone@unina.it

**Keywords:** fibroblasts, breast cancer associated fibroblasts (BCAFs), mammary gland, breast cancer microenvironment, ECM remodeling, metastasis

## Abstract

The stromal microenvironment regulates mammary gland development and tumorigenesis. In normal mammary glands, the stromal microenvironment encompasses the ducts and contains fibroblasts, the main regulators of branching morphogenesis. Understanding the way fibroblast signaling pathways regulate mammary gland development may offer insights into the mechanisms of breast cancer (BC) biology. In fact, the unregulated mammary fibroblast signaling pathways, associated with alterations in extracellular matrix (ECM) remodeling and branching morphogenesis, drive breast cancer microenvironment (BCM) remodeling and cancer growth. The BCM comprises a very heterogeneous tissue containing non-cancer stromal cells, namely, breast cancer-associated fibroblasts (BCAFs), which represent most of the tumor mass. Moreover, the different components of the BCM highly interact with cancer cells, thereby generating a tightly intertwined network. In particular, BC cells activate recruited normal fibroblasts in BCAFs, which, in turn, promote BCM remodeling and metastasis. Thus, comparing the roles of normal fibroblasts and BCAFs in the physiological and metastatic processes, could provide a deeper understanding of the signaling pathways regulating BC dissemination. Here, we review the latest literature describing the structure of the mammary gland and the BCM and summarize the influence of epithelial-mesenchymal transition (EpMT) and autophagy in BC dissemination. Finally, we discuss the roles of fibroblasts and BCAFs in mammary gland development and BCM remodeling, respectively.

## 1. Introduction

The adult mammalian gland develops mainly after birth. It has a complex structure comprising a branching epithelium surrounded by a stromal microenvironment [1]. The interaction between these two compartments, together with a series of growth hormones and growth factors, modulates its development [2,3,4,5]. Notably, in breast cancer (BC), alterations in the signaling pathways regulating the development of the physiological mammary gland contribute to cancer growth [6,7,8,9]. Moreover, mammary stromal fibroblasts, which maintain extracellular matrix (ECM) homeostasis, also modulate morphogenesis in both normal and tumorigenic mammary glands when interacting with epithelial and cancer cells [9,10,11,12]. 

BCs are heterogeneous solid tumors that can be classified into distinctive histological and molecular subtypes, associated with different invasive capabilities, sites of metastasis, and clinical outcomes [12,13,14,15,16,17]. For instance, BCs can express estrogen receptor (ER), progesterone receptor (PR), and human epidermal growth factor receptor 2 (HER-2), whereas those with the worst prognosis, the so called triple negative breast cancers (TNBCs), lack the expression of the three receptors [12].

Like most solid tumors, BCs are very heterogeneous and abnormal tissues, characterized by a stromal tumor microenvironment (TME) that supports tumor development and dissemination [18,19,20,21]. Major players in the structure of the TME and in the behavior of both stromal and cancerous cells are breast cancer-associated fibroblasts (BCAFs). Indeed, these non-cancerous stromal cells represent up to 80% of the tumor mass [12,15]. Not surprisingly, several studies have demonstrated that the recruitment and activation of BCAFs induce deep changes to the TME, thereby sustaining cancer dissemination [15,18,22,23,24]. Of note, El-Ashry’s group [25] showed that BCAFs and cancer cell aggregates circulate in the peripheral blood of patients with metastatic BC. Consistently, additional evidence has demonstrated that they facilitate metastasis by contributing to the formation of metastatic niches in distant organs, thereby facilitating the metastatic process [25,26]. These findings clearly suggest using BCAFs as key diagnostic biomarkers in metastatic BC [25,26]. Considering that BCAFs originate from normal fibroblasts [12], and that both fibroblast types regulate normal and tumorigenic mammary gland development, we believe that a better understanding of the role of fibroblasts and BCAFs in mammary gland and breast cancer microenvironment (BCM) remodeling could contribute to the development of new therapeutic strategies targeting BC growth. Hence, the aim of this paper is twofold. Firstly, we review the most recent findings on the structure of the mammary gland and BCM, and on the influence of epithelial-mesenchymal transition (EpMT) and autophagy in BC dissemination. Secondly, we compare the roles of fibroblasts and BCAFs in regulating mammary gland development and microenvironment remodeling linked to cancer cell dissemination.

## 2. Mammary Gland Structure and Development

**Figure 1:**
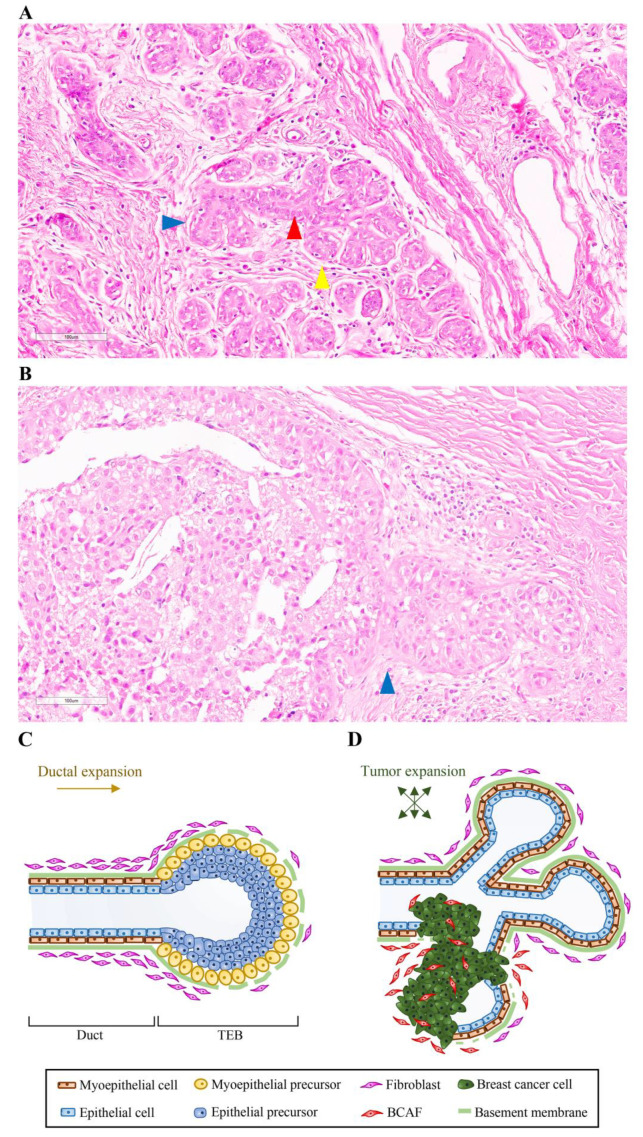
(**A,B**) Haematoxylin and eosin staining. (**A**) Normal structure of human terminal duct lobular units (TDLUs), composed of an inner layer of luminal epithelial cells (red arrow), and an outer layer of myoepithelial cell (yellow arrow), separated from the stroma by a basement membrane (blue arrow). (**B**) Early cancerization of a human mammary duct with initial stromal invasion. Magnification X 20. Whole Slide Imaging were digitized with an Aperio AT2 scanner with 40x optics. (**C,D**) Schematic representations of (**C**) terminal end bud (TEB) during mammary branching morphogenesis and (**D**) ductal and alveolar structure during breast cancer (BC). (**C**) The figure shows the structure of TEB which consists of inner layers of epithelial precursors, also known as body cells, surrounded by an outer layer of myoepithelial precursors, also known as cap cells. A basement membrane, lining the neck of the TEB and the subtending duct, separates the epithelium and the stroma. In front of the expanding TEB, the basement membrane is transiently disrupted, to allow epithelial growth and ductal expansion. Fibroblasts, together with adipocytes (not shown), represent the major cellular component of the mammary stroma, and play a fundamental role in mammary gland development. Indeed, they sustain mammary gland formation by promoting epithelial cell expansion, normal duct elongation, and invasion into the fat pad. (**D**) BC cells break the normal architecture of myoepithelial and epithelial cells in the ducts and alveoli of mammary gland. Epithelial cells can undergo malignant transformation. Myoepithelial cells surrounding the neoplastic lesion disappear. Fibroblasts become constitutively activated in breast cancer-associated fibroblasts (BCAFs), and the basement membrane gradually thins out. BCAFs sustain BC growth and progression by enhancing epithelial tumor cell proliferation, migration, and invasion by paracrine interactions. These events lead to BC expansion inside and outside the mammary gland.

The parenchyma of the adult female mammary gland is composed of tree-like branching ducts spreading radially from the nipple and terminating in expanded alveolar aggregates, known as lobules [4,27]. The duct wall is constituted by an outer and inner layer (Figure 1A,C) [4,27].

The outer “basal” layer is composed of myoepithelial cells connected directly to the basement membrane and displays smooth muscle-like contractile properties. The inner “luminal” layer, instead, includes cells with significantly different, polarized epithelial features. The myoepithelial cell layer, whose contraction sustains milk secretion, regulates the homeostasis of the basement membrane, which is a thin and dense structure made up of collagen IV, laminin, and proteoglycans [28]. This tissue network is embedded in a stromal microenvironment, which includes the extracellular matrix (ECM), blood and lymphatic vessels, and multiple stromal cell types, including fibroblasts, endothelial cells, adipocytes, immune cells, and mesenchymal stem cells (MSCs) [1,3,27]. The ECM contains fibrillar collagen, proteoglycans, glycoproteins tenascin C (TNC), and fibronectin (FN) [29]. Its main function is to regulate mammary cell pathways through cell-cell interactions and paracrine and mechanical signals [28]. The key fibrillar protein in the ECM [3] is collagen I. Collagen I and collagen bundle alignments are important regulators of mammary gland development and duct outgrowth during puberty [30,31]. However, on mammography, a dense breast tissue is associated with abnormal accumulation of collagen I in the breast stroma—an event highly suggestive of cancer development. Indeed, studies have shown that BC cells frequently originates from dense breast tissues and extended beyond the basement membrane into adjacent tissue [28] (Figure 1B,D).

Although mouse and human mammary glands display structural and endocrine differences, the current knowledge of mammary gland development is based on studies in murine experimental models. The reason is that, being easy to handle, they are particularly suited for studying endocrine regulation and stromal epithelial cell crosstalk [1,3,8,27].

By and large, the mammary gland develops mainly postnatally in puberty, pregnancy, lactation, and involution—stages in which reproductive hormones and growth factors play a decisive role [3,4,5]. At the onset of puberty, the ovarian steroid hormones regulate branching morphogenesis by promoting the extension and branching of mammary ducts (Figure 1C) [5]. Then, at a later stage, the epithelium infiltrates the fat-pad stroma and builds the mammary gland. Thereafter, the gland continues to evolve by undergoing differentiation, and de-differentiation during menstruation, pregnancy, lactation, and involution [32]. Outgrowth of mammary ducts begins at the tips of the ducts, namely, the terminal end buds (TEBs). TEBs are mammary structures characterized by a bulb shape. They develop at the onset of puberty under the influence of ovarian estrogen and the pituitary growth hormone. It is in these structures that proliferating cells differentiate into luminal epithelial cells and basal myoepithelial cells, and luminal cell apoptosis generates the hollow ducts (Figure 1C) [33,34,35]. Their function is to modulate ductal growth throughout the fat pad, while undergoing regular bifurcation events, which ultimately lead to the formation of the mammary ductal tree (Figure 1C) [35]. During pregnancy, intense epithelial cell proliferation leads to further ductal branching and alveolar formation. Subsequently, after parturition, the luminal epithelial cells of the inner layer of the alveoli differentiate into secretory cells, thereby eliciting milk production and secretion through the ducts during lactation [4]. Throughout this process, the nuclear steroid receptors ERα and PR regulate the physiological development of the mammary gland [36]. Indeed, studies on ERα and PR knockout mice show that ERα expression in the mammary epithelium is essential for ductal branching and elongation, whereas PR expression in the epithelium is essential for normal secretory alveolar development [37,38].

Another essential player in milk production is the terminal duct lobular units (TDLUs), that is, clusters of multiple small acini located at the distal ends of the ducts [33,39] (Figure 1A). One of the least static structures within the mammary gland, TDLUs constitute a potential site of origin of BC [39,40]. More specifically, in a normal breast, with the conclusion of childbearing and advancing years, TDLUs undergo an involution process linked to reduced numbers of both acini/TDLU and TDLUs [41,42]. Such involution is correlated with age, menopausal status, and parity [43]. On the other hand, the absence of TDLU involution is correlated with an increased risk of BC [44]. Consistently, evidence shows that BC originates in the TDLUs and advances through a series of molecular events that ultimately lead to invasive and metastatic carcinomas. These include increased epithelial cell proliferation, atypical hyperplasia, and carcinoma in situ [33].

### Influence of Fibroblasts on Mammary Gland Development

Research on the role of fibroblasts in the development of the mammary gland is facilitated in mice, because the epithelial branching of the mouse mammary gland continues to develop many weeks after birth [8,9,45]. Two different fibroblast cell populations have been identified in the stroma of the mammary gland: lobular and interlobular fibroblasts [40]. Lobular fibroblasts envelop TDLUs and express high levels of the CD105 marker. Their primary function is to sustain luminal epithelial growth and branching morphogenesis by producing ECM proteins, matrix remodeling enzymes, and several growth factors. Instead, the interlobular fibroblasts express high levels of the CD26 marker and are characterized by an immune-related signature [40]. Conversely, the intralobular fibroblasts are characterized by a myofibroblast-related signature that includes the expression of the transforming growth factor (TGF)-β1, TNC, and α-SMA [40]. Moreover, since their expression profile coincides with that of the tumor stoma, some authors have suggested that these fibroblasts represent a reservoir of BCAFs localized to the TDLU [40]. 

The presence of aberrantly activated stromal cells, especially myofibroblasts, can favor BC development and sometimes precedes the malignant transformation of epithelial cells in the mammary gland [12,46,47]. This process, named desmoplasia, not only generates a niche for cancer cells, but also produces a hypoxic and acidic microenvironment responsible for compromising chemotherapeutic treatments [21].

Interestingly, scientists have recently turned their attention to examining the underlying mechanisms by which obesity may predispose women to BC. Several studies suggest that obesity sustains myofibroblast differentiation within the mammary adipose tissue, thereby inducing fibrotic remodeling of the microenvironment and BC development [48,49,50]. Consistently, in mammary tissues of obese mice, myofibroblast differentiation increases the activity of the TGF-β1/SMAD3/miR-140 negative-feedback loop. In particular, the activation of TGF-β1 drives SMAD3 activation that, in turn, inhibits miR-140 transcription by binding to the miR-140 locus. This signaling network impedes SMAD3 degradation by miR-140, thus promoting myofibroblast differentiation and fibrosis [1]. Furthermore, mammary stromal vascular fraction from high-fat diet–fed mice has been shown to induce self-renewal and the invasive capability of the non-invasive BC cell line MCF10DCIS [1]. 

On the other hand, myofibroblasts also regulate the morphogenesis of normal breast epithelial cells. In fact, 3D-collagen I cocultures of non-malignant human breast epithelial cell line HMT-3522 and immortalized human mammary myofibroblasts lead to the formation of acinus-like structures characterized by a central lumen and cell growth arrest [11]. More specifically, myofibroblasts induce epithelial cell differentiation by affecting matrix tension, and by interacting with paracrine signaling activity [11]. Conversely, 3D-collagen I monocultures of HMT-3522 cells drive uncontrolled cell proliferation and morphological disorganization, resembling a pattern of BC in vivo [11].

**Figure 2:**
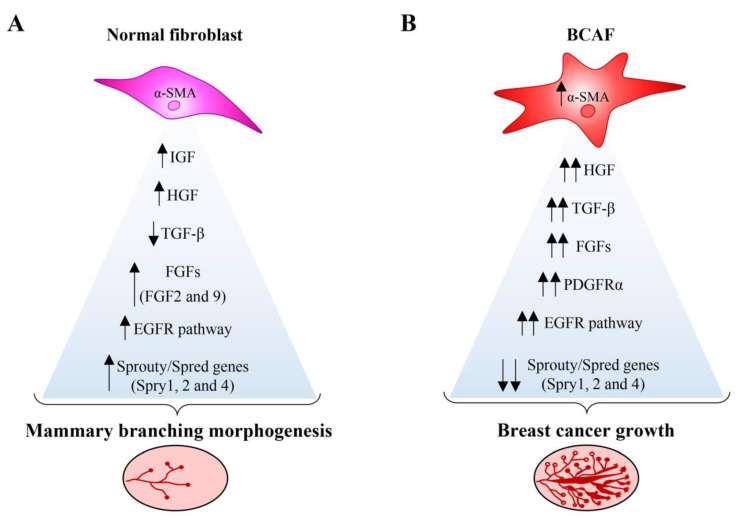
Role of fibroblasts in mammary gland branching and BC growth. Mammary branching morphogenesis and BC growth share many cellular processes, including epithelial cell proliferation, migration, invasion, and extracellular matrix (ECM) remodeling. These similarities explain why many morphogenic processes induced by fibroblasts are exploited intensively also in BC. (**A**) In normal conditions, fibroblasts guide mammary epithelial branching through paracrine signals that are mediated by growth factors, such as insulin-like growth factor (IGF), hepatocyte growth factor (HGF), fibroblast growth factor (FGF)2, and FGF9, low concentrations of transforming growth factor (TGF)-β and tightly regulated activation of the epithelial growth factor receptor (EGFR) signaling pathway. The expression of Sprouty/Spred family members in stromal cells plays a very important role during mammary branching. These morphogenic processes activated by fibroblasts are tightly controlled to ensure normal mammary gland development. (**B**) However, they become dysregulated and subverted during BC. In fact, BCAFs support BC growth by increasing extensively the same morphogenic pathways required for normal gland development. BCAFs massively upregulate the platelet derived growth factor receptor (PDGFR)α and EGFR signaling pathways, and produce higher levels of FGFs, HGF, and TGF-β than normal fibroblasts. The loss of Spry genes promotes, in fibroblasts, a cancer-associated fibroblast (CAF)-like phenotype and thus leads to BC growth.

Stromal fibroblasts also regulate branching morphogenesis and ECM remodeling during the different stages of mammary gland development [51,52,53,54] by interacting with several signaling pathways. For instance, during epithelial branching, normal fibroblasts work alongside paracrine signals, which are mediated by a series of growth factors, including fibroblast growth factors (FGFs), insulin-like growth factor (IGF), hepatocyte growth factor (HGF), and TGF-β1 [55,56] (Figure 2A). 

Under normal conditions, the FGF or IGF signaling activity is reduced, stunting epithelial branching. Conversely, when FGF is upregulated, cell polarity is disrupted, thereby inducing cell proliferation, cell survival, and invasion capability. As shown in Figure 2B, this chain of events closely mirrors the molecular processes occurring during BC development [57,58,59]. Low concentrations of TGF-β1 also contribute to mammary ductal morphogenesis mediated by fibroblasts (Figure 2A) [56]. Moreover, FGF signaling, elicited by FGF2 and FGF9, triggers ERK1/ERK2 activation, cell proliferation, and migration in the murine mammary gland. FGF2 is also involved in fibroblast collagen remodeling and modulates fibroblast ECM synthesis. Evidence shows that FGF2 and FGF9 signaling in primary mammary fibroblasts, co-cultured with 3D epithelial organoids, sustains the branching of the mammary epithelium (Figure 2A) [60]. Moreover, the epithelial growth factor receptor (EGFR) pathway also influences mammary gland stroma (Figure 2A) [61]. In brief, the activation of the EGFR pathway in stromal cells, which are localized in front of the invading TEB, stimulates matrix metalloproteinase (MMP)-14 expression and MMP-2 activation—a process leading to ECM remodeling and epithelial branching [61].

Different members of the Sprouty/Spred gene family, which are considered tumor suppressor genes, also participate in mammary branching morphogenesis by interfering with EGFR signaling activity [9,62]. Among these is Spry 1. In vitro and in vivo analysis show that loss of Spry1 function in mammary fibroblasts drives upregulation of EGFR signaling, following amphiregulin and TGF-α stimulation, consequently increasing ECM remodeling and mammary epithelium branching in pubertal mice. Another member of this gene family participating in mammary branching morphogenesis is Spry2. Spry 2, expressed in the luminal epithelial, myoepithelial, and stromal cells of the mouse mammary gland, also affects FGF signaling associated with physiological epithelial morphogenesis (Figure 2) [63] Conversely, gain of Spry2 function in mammary fibroblasts reduces EGFR signaling associated with stunted epithelial branching [9]. Using quantitative phosphoproteomic analysis, Shi et al. showed that deletion of Spry1, 2, and 4 in triple-knockout (SpryKO) mouse mammary fibroblasts, triggers a BCAF-like phenotype associated with alteration in ErbB, insulin, vascular endothelial growth factor (VEGF), focal adhesion, and mTOR signaling pathways [64]. Therefore, the loss of fine regulation of Spry/EGFR signaling alters mammary gland development and drives BC development by increasing the invasive capability of tumor cells [9,64] (Figure 2B). This evidence clearly suggests that the components of the EGFR family constitute potential therapeutic targets [65].

Platelet derived growth factor receptor (PDGFR) α activity induces activation of quiescent fibroblasts and ECM remodeling [66]. Indeed, the constitutive activation of PDGFRα in mice mammary fibroblasts hinders the invasion and branching of the ductal epithelium during post-pubertal development. However, it does not alter the epithelial differentiation program, and elicits fibrosis and a pro-tumorigenic stromal microenvironment (Figure 2) [67]. In addition, PDGFRα^+^ fibroblasts from involuting mice mammary glands, display an activated phenotype, COX/prostaglandin E2 (PGE2) dependent activity, and pro-tumorigenic and immunosuppressive activity in an immunocompetent and orthotopic tumor transplant model [68]. Moreover, PDGFRα^+^ mammary fibroblasts from involuting mammary gland show increased expression levels of fibrillar collagens, TGF-β1, Lysyl oxidase (LOX), MMP-2, MMP-3, and CXCL12 genes, compared to mammary fibroblasts from nulliparous mouse mammary glands. Accordingly, PDGFRα+ mammary fibroblasts could be potential targets for the prevention and treatment of postpartum BC; notably, the nonsteroidal anti-inflammatory drug (NSAID) ibuprofen has been shown to suppress their pro-tumorigenic activity [68]. 

SHANK-associated RH domain interacting protein (SHARPIN) is a ubiquitin-related protein involved in several processes, including inflammatory responses, the development of both normal and cancer tissues [69], and inhibition of integrin activity in different cell types [70,71,72,73]. Under physiological conditions, stromal SHARPIN regulates ductal outgrowth during puberty [32]. In particular, SHARPIN modulates ECM remodeling during mammary gland branching morphogenesis [32]. Furthermore, the SHARPIN/α11β1 axis regulates mechano-transduction in mammary gland fibroblasts [74]. 

Notably, progress on the current understanding of the interaction between the epithelium and stroma during mammary gland, and BC development has been made possible thanks to the implementation of the cleared fat-pad transplantation experimental system [75]. Today, it is possible to mimic a normal or malignant human breast tissues in mice by resecting the epithelium from the fat pad of the mammary gland within just three weeks after birth, by inoculating human stromal fibroblasts, as well as a mixture of human mammary fibroblasts and epithelial cells [76,77,78]. Remarkably, the inoculated human fibroblasts sustain the engraftment of the subsequently injected mixture of human mammary fibroblasts and epithelial cells. Based on this evidence, it has been further confirmed that the mammary stroma provides the proper environment for the development of either normal mammary epithelium or benign and malignant breast lesions [76].

## 3. Hallmarks of Metastatic BCM

As opposed to breast carcinomas in situ, invasive breast carcinomas have the capability to invade the mammary stroma, vascularize, and metastasize [33,79]. The invasive and capability of cancer cells is dramatically influenced by the TME, which consists of non-cancer stromal cells, blood vessels, lymphatic tumor vessels, and ECM [12,14,18,21,26]. Non-cancer stromal cells include BCAFs, MSCs, adipocytes, T cells, natural killers, macrophages, endothelial cells, and pericytes, the last of which participate in blood and lymphatic vessel formation [15]. In particular, BCAFs are very heterogeneous and abundant non-cancerous cell types of the BC stroma, and dramatically contribute to cancer cell metastasis [12,15,80]. BCAFs mainly derive from normal resident fibroblasts, which are induced by cancer cells to enter into a constitutively activated state [12]. Approximately 80% of normal breast fibroblasts can be reprogrammed into BCAFs through a complex cascade of events [50,81,82,83]. Generally, by continuously interacting with cancer cells, normal fibroblasts enter into what is known as a reversible “primed state”. In this initial state, primed fibroblasts are already able to sustain the progression of cancer despite not yet expressing all the features of constitutively activated BCAFs [12,84]. Subsequently, they activate and enhance several autocrine signaling pathways, which enable them to preserve the myofibroblastic phenotype and acquire cancer-associated fibroblast (CAF) phenotype even in the absence of the paracrine signaling of BC cells [12].

In solid tumors, the crosstalk between cancer cells and fibroblasts is modulated by reactive oxygen species (ROS), which drive CAF differentiation and constitutive activation [21]. Among them, hydrogen peroxide (H_2_O_2_) is the main ROS regulating the signaling pathways of normal and cancer cells and supporting CAF differentiation [18,85,86]. However, ROS overproduction can inhibit tumor growth and lead to cancer cell death. At early stages of cancer progression, transformed epithelial cells generate a reductive tumor microenvironment by decreasing oxidative stress and by upregulating endogenous antioxidant defenses, thereby promoting tumorigenesis [87]. Therefore, targeting endogenous antioxidant defenses by using pro-oxidant molecules, like 1,25-dihydroxyvitamin D, could represent a promising therapeutic strategy for cancer prevention [88]. In support of this hypothesis, Wilmansk et al. demonstrated that 1,25-dihydroxyvitamin D enhances oxidative stress in early BC progression and decreases BC cell viability [88].

It is known that, when BCAFs interact with cancer cells, they produce high levels of TGF-β [84], which is involved in myofibroblast [89] and CAF differentiation [90,91,92]. BCAFs share similarities with activated fibroblasts, named myofibroblasts, i.e., cells involved in wound healing and inflammatory processes [12]. However, unlike normal myofibroblasts, BCAFs fail to undergo apoptosis [12]. They persist in the BC stroma by maintaining a constitutive myofibroblastic phenotype associated with α-SMA expression. This protein, which is the most significant marker of fibroblast activation and myofibroblast differentiation [93], is highly expressed in BCAFs. For this reason, it is widely used as a CAF marker (Figure 2B). Other important markers for the identification and characterization of BCAFs are vimentin, fibroblast specific protein (FSP), fibroblast activation protein (FAP), osteonectin, desmin, TNC, PDGFR α/β, and the loss of Cav-1 and/or CD34 [12,94,95].

**Figure 3:**
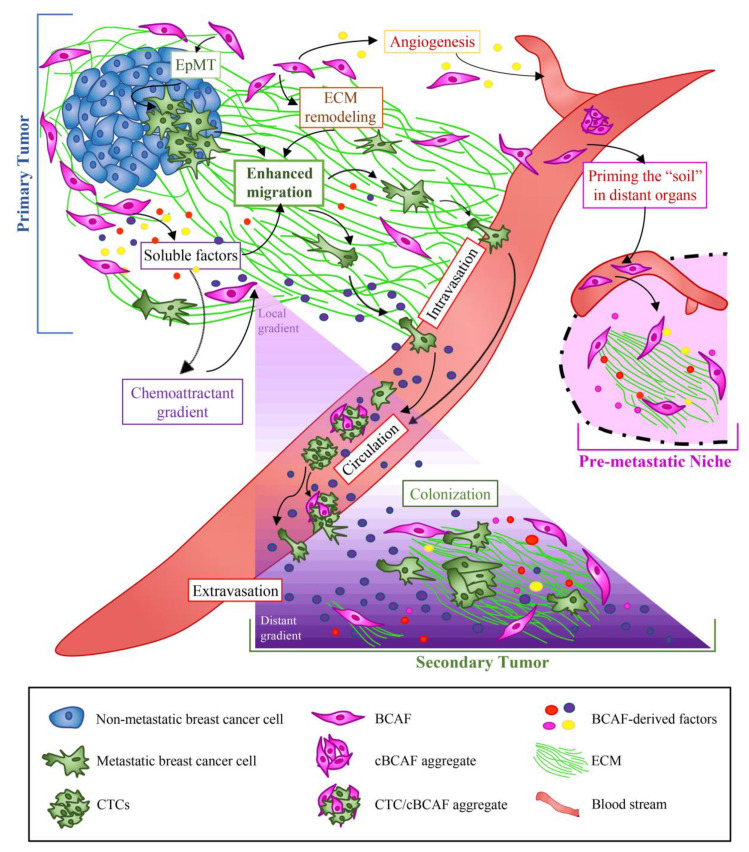
Role of BCAFs in BC metastasis. BCAFs can create favorable physical conditions for BC metastasis. Through ECM remodeling, enhanced and aberrant angiogenesis, soluble factor release and establishment of local and distant chemoattractant gradients, BCAFs sustain and enhance BC cell migration, intravasation in blood circulation (as circulating tumor cells (CTCs), CTC aggregate and/or CTC/BCAF aggregate), extravasation, and, ultimately, the homing and colonization of secondary sites. Additionally, through paracrine interactions, BCAFs promote epithelial-mesenchymal transition (EpMT), and the activation of several signaling pathways in BC cells. All of these processes enhance the capability of BC cells to migrate, produce proteases, enter the blood circulation and finally colonize a secondary tumor site. Notably, BCAFs can disseminate through the blood circulation as cBCAFs and/or aggregates without cancer cells, colonize and modify the “soil” in distant organs, thereby creating a receptive microenvironment (the pre-metastatic niche) for distant tumor growth. The color gradient (from lighter to darker violet), starting in the primary tumor, proceeding in the blood stream, and ending in the secondary tumor, indicates the gradual increase of BCAF-derived chemoattractive factors. Lighter colors depict lower concentrations. Darker colors depict higher concentrations.

BCAFs are also involved in ECM synthesis and remodeling, and support BC cell migration, invasion, and survival in blood circulation [15,25]. In this complicated network, breast cancer cells, by paracrine interactions, recruit and activate stromal cells that are involved in all steps of the metastatic process (Figure 3) [15,96,97].

The detachment of cancer cells from tumor nests and epithelial-mesenchymal transition (EpMT) constitute the first steps of the whole metastatic process (Figure 3) [98,99,100]. Practically, cancer cells invade the neighboring tissue, cross the vascular endothelial layer, and enter the blood and lymphatic vessels, thus becoming circulating BC cells. The metastatic process is completed by extravasation of metastatic cancer cells and formation of metastasis at organ sites distant from the site of the primary tumor (Figure 3) [100]. 

In this multistep-process, lymphatic and blood vessels play an essential role in supporting cancer cell dissemination and metastasis. Their formation occurs in the TME through the activation of paracrine signals deriving from both cancer and stromal cells [100,101,102]. Lymphatic vessels are more permeable than blood vessels, so they have long been considered the preferential route of BC cell dissemination [103]. However, some studies have contradicted this hypothesis. For instance, a recent work analyzing the blood and lymphatic vasculature in primary BC and lymph node metastases has demonstrated that, whereas blood vessels always localize within tumor cell nests (TCNs) and tumor-associated stroma (TAS), lymphatic vessels rarely do so. Indeed, they tend to localize mainly between cancer cells and stromal cells [104].

Other important players in the metastatic process are specific intravasation sites termed tumor microenvironment of metastasis (TMEM) [105]. In particular, these specific microanatomical structures act as “doorways”, through which migrating cells enter into the bloodstream. They include perivascular macrophages, vascular endothelial cells, as well as cancer cells expressing high levels of the mammalian-enabled (Mena) isoform. This actin-regulatory protein elicits cell movement, invasion, and EGF-induced metastasis. The concerted action of these cells forms a micro-anatomic structure that enables migrating cancer cells to enter into the circulation, eventually determining the severity of the metastatic outcome in BC patients [106,107,108]. Tumor cell intravasation happens at TMEM sites where VEGF, secreted by TMEM macrophages, triggers a momentary blood vessel permeability [109]. Importantly, since TMEM has been detected in primary human BCs, in both the regional lymph node and distant metastases [106], it constitutes an essential metastatic biomarker for predicting early recurrence within the first 5 years after diagnosis, as evidenced in a uniformly treated clinical trial cohort of patients with ER^+^/PR^+^/HER2^−^ early stage BC [110]. 

The ECM plays a pivotal role in the invasive capability and metastatic cascade. Fundamentally, it acts as a functional scaffold that provides biophysical and biochemical cues to influence cell adhesion, migration, and dissemination [111]. The intertwined structure of the ECM is made of different molecules, such as collagen, laminin, FN, proteoglycans, and tenascin [15,21,112,113]. Additionally, it stores molecules and growth factors, such as MMPs, protease inhibitors, and VEGF, all of which regulate the metastatic process [21,100]. Kim et al. reported, for instance, that high levels of VEGF cause BC cells to form brain metastases [114]. In fact, by using a BC mouse model, they demonstrated that the VEGF-receptor tyrosine kinase inhibitor PTK787/Z 222,584 significantly reduces CD31+ vessels in brain lesions, enhances apoptosis within the tumor mass, thereby decreasing the tumor burden in the brain [114]. This is in line with evidence showing that CD31 expression is often upregulated in endothelial cells of brain metastases of BCs, cervical carcinomas, and non-small cell lung cancers [115,116]. 

Among the various molecular components of the ECM responsible for BC malignancy is collagen I. BC tissue is characterized by a desmoplastic microenvironment. Such a microenvironment is mainly due to collagen deposition and cross-linking associated with ECM stiffening, growth factor signaling, and, ultimately breast malignancy [117]. Indeed, studies have shown that increased expression of collagen I is associated with a high occurrence of metastasis [118].

Fundamentally, the passage from non-malignant breast tissue to invasive ductal carcinoma (IDC) is significantly associated with deposition, linearization, and bundling of collagen. In fact, accumulation of collagen fibers drives ECM stiffening that, in turn, activates mechanically sensitive signaling pathways regulating focal adhesion, mechanically-activated factor Yorkie Associated Protein (YAP), TGF−β signaling, and pro-tumorigenic macrophage infiltration [119]. Furthermore, cross-linking and collagen bundling, regulated by the cross-linking enzymatic activity of LOX, create the molecular tracks that pave the way for cancer cells to migrate beyond the primary tumor [117,120,121,122]. Indeed, studies have shown that high expression of LOX in BCAFs induces ECM remodeling, invasion and metastasis of MDA-MB-231 cells in vitro and vivo experiments [123]. The role of LOX in the early stages of metastasis has been well established in the literature. It is produced and secreted in the vascular network by the tumor hypoxic microenvironment by virtue of the generation of extending avascular regions, structural/functional vessels alterations, and upregulation of hypoxia-inducible factors (HIFs) [124,125,126,127,128,129]. When this cross-linking enzyme reaches the bones of BC patients, it alters tissue homeostasis through bone remodeling, thus generating pre-metastatic bone lesions in ER^-^ BC patients. Subsequently, these lesions provide a scaffold for circulating BC cells to generate bone metastases [130]. In fact, the pre-metastatic niche is a favorable, permissive and hospitable microenvironment required for tumor engraftment (Figure 3). As shown in the Figure 3, the pre-metastatic niche evolves into a metastatic niche after the homing and the engraftment of circulating tumor cells (CTCs) [131]. Notably, LOX could be a potential therapeutic target for invasive BC, since selective LOX inhibition reduces angiogenesis, BCAF activation, and tumor growth [132,133]. Damaging the collagen network could hinder cancer cell metastatic capability and improve therapeutic strategies [134]. Moreover, the analysis of the collagen network with non-invasive magnetic resonance elastography could provide significant images to assess collagen secretion and deposition modulated in the ECM by antifibrotic therapeutic strategies [135]. 

Laminins are expressed in the basal epithelium and form the basement membrane [136]. Paracrine signals and the direct interaction between invasive MDA-MB-231 cells and myofibroblasts from the IDC interface zone induce laminin 332 (LN332) upregulation and neo-expression of β4 integrin in myofibroblasts, respectively [137]. The coexpression of LN332 and integrin β4 elicits myofibroblast resistance to anoikis. Therefore, the anoikis resistant phenotype makes myofibroblasts, localized to the interface zone, the principal cells implicated in tissue remodeling during BC cell invasion [137].

The role of FN in tumorigenesis is still controversial [138]. However, FN expression in primary BCM is inversely correlated with patient survival, and FN production is upregulated after EpMT and during late stage of BC [139,140]. Furthermore, the polymerization and organization of microenvironmental FN are essential for the accumulation of collagen I. FN-collagen interaction is mediated by MMPs that remodel the ECM, and alter pro-angiogenic signaling associated with BC invasiveness [141]. Therefore, owing to the paradoxical role of FN in tumor progression, further studies are warranted to elucidate whether it can actually constitute a potential therapeutic target. 

Another important component of the ECM is Syndecan 1 (Sdc-1). It is a cell surface heparan sulfate proteoglycan that is aberrantly expressed by BCAFs in more than 70% of human BCs [142]; particularly, it is highly upregulated in the stromal compartment of IDC [143]. Sdc-1-expressing BCAFs generate an abnormal and permissive ECM for BC cell migration and invasion. In particular, ECM deposited by Sdc-1^+^ BCAFs contains high levels of FN and displays a well-organized and parallel fiber organization that promotes BC cell attachment, invasion, and directional migration [144]. 

Finally, isoforms of the extracellular glycoprotein tenascin (TNC) also contribute to the metastatic capability of BC cells [15]. TNC is more expressed in breast ductal carcinomas than in normal breast tissues. Therefore, its expression could represent a prognostic and BCAF marker for breast ductal carcinoma [145]. For example, one of its isoforms, namely, Tenascin W (TNW), has been detected in the serum of BC patients and in the BC stroma, thus making it a BC biomarker [146]. Furthermore, experiments in vitro show that TNW supports fibroblast adhesion and induces cancer cell migration toward FN [147].

## 4. Influence of Epithelial-Mesenchymal Transition and Autophagy on BC Metastasis and Dissemination

EpMT is a biological process that allows epithelial cells to undergo a morphological reprogramming of cellular architecture and acquire mesenchymal features. Such a phenotypic change, supported by a massive reorganization of the cytoskeletal filaments, affects several features, which include cell adhesion [148], polarity [149], motility, and morphology [150], and is mainly responsible for induction of metastatic potential [151], drug and apoptosis resistance [152], and cancer progression [153].

EpMT enables tumor cells to leave the primary tumor environment, enter the bloodstream, and disseminate [154,155]. Then, during the outgrowth of metastatic tumors, cancer cells return to an epithelial state by undergoing mesenchymal-epithelial transition (MET). This process enhances the cells’ capability to overcome dormancy, undergo metastatic outgrowth, and adapt to a new microenvironment [155]. Furthermore, epithelial-mesenchymal plasticity (EMP) acts as a driving force for metastatic cancer by efficiently facilitating the completion of the steps involved in the metastatic cascade. In particular, whereas EpMT helps tumor cells to initiate the metastatic cascade, MET helps disseminated cancer cells to complete the metastatic process by forming macro-metastasis at distal organs [154,155,156]. 

EpMT-like morphological changes are also promoted by the fibrillar FN matrix, which supports the proliferation and migration of metastatic BC cells. However, the BC cells that constitutively express FN maintain an extremely stable mesenchymal phenotype that limits their metastatic potential. Despite not being competent for metastasis themselves, constitutively mesenchymal tumor cells still contribute to the metastatic progression by facilitating migration and invasion of metastatic-competent tumor cells in a paracrine manner. In this scenario, FN-expressing cancer cells act in a stromal fashion, to facilitate the growth of metastatic tumor cells [154]. Moreover, FN is expressed transiently during the different stages of the metastatic cascade. EpMT enhances intracellular FN levels to support cell migration and dissemination [157]. Conversely, inhibition of FN enables mesenchymal BC cells to complete MET and form secondary tumors within the metastatic microenvironment [154,157]. Equally interesting is that the orientation of FN and collagen fibrils changes during tumor progression. In particular, studies show that during premetastatic growth, FN and collagen fibrils are oriented parallel to the primary tumor edge. Conversely, in invasive tumors, these fibrils are perpendicular to the tumor border, acting as “tracks” for cancer cell migration across the basement membrane [157]. 

The cross-linking enzyme transglutaminase-2 (TG-2), in association with FN, is an EMP marker. Indeed, its upregulation within the primary tumor correlates with metastatic progression. In particular, TG-2 and fibrillar FN are upregulated on the extracellular vesicles (EVs) derived from metastatic BC cells that have undergone EpMT/MET. These metastatic BC cells use TG-2 to educate pulmonary fibroblasts to generate a metastasis-supportive pulmonary niche [155], characterized by enhanced FN matrix accumulation [157]. The accumulation of FN deposited by fibroblasts or fibroblast-like cells makes it possible to predict the exact site of metastatic formation [158]. Notably, TG-2 inhibition can limit BC progression by preventing local fibrotic reactions, and by blocking the formation of the metastatic niche [155]. 

EpMT and autophagy are two fundamental biological processes in cancer, and are strictly linked to each other. Under physiological conditions, autophagy mediates degradation of harmful or dysfunctional cellular components, including pathogens, senescent proteins, and organelles, and modulates the immune response to several stimuli by regulating specific immune processes including antigen presentation, cell activation, and differentiation [159,160,161,162]. Moreover, whereas in normal tissue autophagy also plays an onco-suppressive role, in cancer tissue it promotes the survival and growth of cancer cells. In particular, it is commonly accepted that autophagy plays an anti-oncogenic role in early tumors but has a pro-tumoral role in established cancers [163]. In normal cells, autophagy contributes to the physiological energetic and metabolic homeostasis through the autophagy-dependent clearance of aged and damaged mitochondria and other organelles, thus preventing the activation of the typical metabolic switches of cancer cells [164]. Moreover, autophagy avoids the accumulation of genotoxic molecules and contributes to maintaining genomic stability [165]. However, in established tumors, autophagy is exploited by cancer cells to overcome both intracellular and environmental stress [166], such as nutritional restrictions, hypoxic environment, drugs, overproduction of ROS, and nitrogen species (NOS) by dysfunctional mitochondria [166] and protein aggregates caused by the increase of translation rate [167]. In this regard, several papers have shown a correlation between autophagy dysregulation and cancer [168,169,170]. 

Consistently, recent works have indicated that autophagy plays an essential role in regulating EpMT in cancer, although many results are controversial and limited to a specific tumor, or to a precise stage of tumor progression.

Akalay et al. observed that the BC cell line MCF7 with an EpMT phenotype is less susceptible to T-cell cytotoxicity (CTL) when autophagy is induced. Specifically, Beclin-1 (BECN1) downregulation by RNA interference, and the consequent block of autophagic flux, rescue cells from susceptibility to CTL, suggesting that autophagy plays a pivotal role in supporting cancer cells with an EpMT phenotype, to evade the immunosurveillance process during tumor spreading [171].

In breast and head and neck cancers, IL-6-STAT3 signaling is a strong inducer of EpMT process and causes a massive increase of TWIST and SNAIL gene expression, important EpMT markers. In addition to the role of the STAT3 pathway in response to stress, STAT3 is also able to inhibit autophagy. It was reported that STAT3 binds to and inhibits protein kinase R (PKR), preventing the phosphorylation of eIF2α, an important event of autophagy induction, consequently limiting the autophagic flux [172,173,174]. 

In human BC, NF-κB signaling is essential for the transcriptional activation of genes involved in the EpMT process, such as Twist, Snail1, Slug, all of which contribute to cancer progression [175,176].

It was recently reported that autophagy inhibition in RAS-mutated cells induces EpMT, and this process is partly due to NF-κB induction via the autophagosome cargo SQSTM1/p62 [177]. Thus, in this context, autophagy activators might be used to inhibit NF-κB pathway and to repress EpMT.

Another signaling pathway converging on the modulation of EpMT and autophagy is represented by TGF-β. In particular, during the early phases of tumorigenesis, TGF-β-mediated autophagy represents a TGF-β-dependent tumor suppressive program, whereas, during the late stages of cancer, TGF-β represses autophagy and induces EpMT, thus sustaining the metastatic spreading of cancer cells [178]. The interplay between TGF-β and autophagy is also based on their ability to regulate EpMT through the induction and clearance of ribonucleoprotein complexes, known as processing bodies (P-bodies), which are involved in post-transcriptional mRNA metabolism in cells exposed to various stress stimuli [179]. Interestingly, Hardy et al. reported that in mammary epithelial cells, the formation of P-bodies is a critical step for TGF-β to induce EpMT, whereas the clearance of P-bodies by autophagy blocks EpMT and prevents metastasis formation [179]. During the last step of the metastatic process, i.e., metastatic colonization, cancer cells use autophagy-mediated clearance of P-bodies in order to return to an epithelial phenotype and colonize the metastatic niche successfully. Spleen tyrosine kinase (SYK), present in P-bodies formed during the onset of EpMT, is essential for the autophagy-mediated clearance of P-bodies during MET. The disruption of autophagy by a specific genetic knockout or the specific SYK inhibitor fostamatinib blocks autophagy-mediated clearance of P-bodies and MET, therefore inhibiting metastatic tumor outgrowth [180]. 

Therefore, this compelling evidence clearly suggests that a better understanding of the mechanisms driving EMP and autophagy in BC may be a fundamental step to developing more effective anti-cancer therapies.

## 5. Role of BCAFs in BC Cell Dissemination and Metastasis

Metastasis accounts for more than 90% of BC related deaths. Therefore, an improved understanding of the underlying mechanisms leading to metastasis is of vital importance to provide novel prognostic biomarkers and potential therapeutic strategies to prolong survival, and improve the quality of life of patients with metastatic BC [181]. In the previous sections, we discussed the various molecular mechanisms whereby ECM remodeling spurs cell migration and invasion. In the following sections, we will attempt to address the involvement of BCAFs in promoting BC dissemination and metastasis.

In addition to contributing to the formation and remodeling of the ECM, BCAFs also induce cancer cell migration and invasion by secreting several soluble factors (Figure 3) [182]. Among them are TGF-β [183], HGF, basic fibroblast growth factor (bFGF) [84,184,185,186], FSP-1 [187], CCL11, CXCL14 [188,189], CCL-2 [190], and IL-6 [191]. In relation to soluble factors and paracrine interactions, Xu et al. demonstrated that BCAFs lacking the Tiam1 protein produce high levels of OPN that, in turn, promote EpMT, cancer stem cell phenotype, BC invasion, and metastasis. Interestingly, the authors observed that whereas Tiam1 deficiency in fibroblasts is associated with increased tumor invasion, Tiam1 expression in cancer cells facilitates tumor growth. Therefore, they identified the Tiam1-OPN axis as a specific pathway involved in the induction of BC metastasis. In fact, in vitro and in vivo studies showed that Agelastatin A, a novel small OPN inhibitor, significantly prevents lung metastasis without altering primary tumor growth. Therefore, these data suggest that targeting the fibroblast Tiam1-OPN pathway could represent a promising strategy for preventing BC metastasis [192]. Interestingly, Shinde et al. identified pyruvate carboxylase (PC) as a novel promising therapeutic target for the treatment of BC metastases to the lungs. They demonstrated that PC is specifically required for initiation of secondary BC in the lung, but it is not required for extrapulmonary cancer growth [193].

BACFs also secrete IL-32 and IL-6, both responsible for triggering a cascade of molecular mechanisms leading to metastasis. Upon secretion, IL-32 binds to integrin β3 on the BC cell surface, activating the p38 MAPK signaling pathway, which in turn, drives BC cell invasion [194]. Moreover, the secretion of IL-6 into the tumor mass contributes to a positive feedback loop of growth and invasion between CAFs and breast epithelial cells. Still, IL-6 further mediates a reciprocal growth relationship between CAFs and mast cells. In fact, IL-6 secreted by mast cells induces the proliferation of fibroblasts, which in turn secrete IL-6, thus promoting mast cell proliferation. In this complex scenario, CAF-derived IL-6 sustains tumor growth and invasion via EpMT of breast epithelial cells. Additionally, BC cells acquire an autocrine IL-6 signal that reinforces tumor growth and invasion [195]. Moreover, CAF-derived IL-6 promotes BC cell growth and invasion through induction of Notch-3, Jagged-1, and carbonic anhydrase IX, all of which may represent attractive targets for developing new anti-tumor therapies [196]. Interestingly, Jayatilaka et al. showed that, although IL-6 and IL-8 are both individually required for BC metastasis, only in combination do they promote BC cell migration. The pharmacologically use of neutralizing antibodies targeting IL-6 (Tocilizumab) and IL-8 (Reparixin) receptors has been shown to significantly reduce metastasis to the lungs, liver, and lymph nodes in preclinical BC models [197]. Furthermore, CAFs can promote BC metastasis indirectly by secreting CCL5, which attracts CD4+/FOXP3+ Treg cells involved in BC cell dissemination to the lungs [198].

In addition to the CAF-derived soluble factors, BC metastasis can also be induced by functional delivery of specific stromal factors via fibroblast-derived exosomes into BC cells. In particular, p85α-deficient fibroblasts, which acquire CAF properties, significantly increase the expression of Wnt10b, which, in turn, activates the canonical Wnt signaling pathway in BC cells. Consequently, Wnt/β-catenin signaling promotes EpMT and enhances BC cell migration and metastasis both in vitro and in vivo. Therefore, the tumor suppressor p85α could represent a new potential marker for BC diagnosis, prognosis, and targeted therapy [199]. Additionally, Luga et al. demonstrated that fibroblast-derived exosomes promote BC cell motility and metastasis by activating the autocrine Wnt-planar cell polarity (PCP) signaling pathway in cancer cells [200]. This evidence suggests that therapeutic strategies targeting the paracrine pathways that regulate tumor migration may be potentially effective in decreasing the metastatic capability of BC cells.

### 5.1. Primary, Circulating, and Metastatic BCAFs: The Onset of the Metastatic Voyage

Recent data have demonstrated that BCAFs can spread beyond the primary tumor and move through the bloodstream as circulating BCAFs (cBCAFs) or BCAF aggregates, with or without cancer cells (Figure 3). Clusters of cBCAFs alone and of clusters of circulating tumor cells (CTCs) with cBCAFs have been detected in the peripheral blood of metastatic BC patients. In addition to providing clinical evidence of the association of cBCAFs with metastatic disease, these findings also suggest the diagnostic potential of cBCAFs and CTCs as biomarkers for BC metastasis [25]. Given the presence of CAFs in the pre-metastatic niche before the appearance of cancer cells [201], it is possible to speculate that, at the end of their metastatic voyage through the blood circulation, cBCAFs colonize a new body site and prime the ”soil” for future tumor cell colonization. Furthermore, cBCAFs can also prime the “soil” by establishing and maintaining a gradient of specific chemoattractive factors, which could trigger the directional migration of BC cells and promote dissemination and colonization of the metastatic niche (Figure 3). For example, Farmaki et al. showed that CCL8 production is enhanced in fibroblasts at the margins of breast tumors. Thus, by establishing a CCL8-gradient, CAFs attract cancer cells towards the tumor margins, precisely where the highest amounts of CCL8 are detected. In response to elevated levels of CCL8, BC cells seed the peripheral “soil”, and enter the circulation by establishing CTC populations. In turn, by following the CCL8 gradient in the blood circulation, CTCs establish secondary tumors in the lungs and brain, as evidenced by the presence of elevated levels of CCL8 in both organs [202].

Additionally, in vitro and in vivo studies have shown that BCAFs promote metastasis by inducing the formation of tumor cell clusters. Specifically, BCAF-derived SDF-1 and TGF-β, via Src activation, induce the formation of multicellular clusters composed of two distinct tumor cell populations: one is composed of tumor cells in a highly epithelial (E^hi^) state, as exemplified by the upregulation of the epithelial marker E-cadherin and the oncogenic cell–cell adhesion molecules, CAM5 and CAM6. The other, instead, consists of cancer cells in a hybrid epithelial/mesenchymal (E/M) state, as exemplified by the upregulation of the mesenchymal marker ZEB1. Both populations are fundamental for BC metastasis; in fact, the suppression of either the epithelial markers or ZEB1 reduces metastasis formation. In particular, E^hi^ cancer cells allow the formation of multicellular clusters and metastatic seeding by promoting cell–cell adhesion, aggregation, anti-apoptosis, and cell proliferation. On the other hand, E/M cancer cells act as leader cells that, by maintaining cell-cell interaction with the follower E^hi^ cancer cells, drive their collective cell invasion and metastatic colonization [203]. The potential clinical role of BCAFs in cancer cell cluster formation and metastasis progression is also supported by findings indicating the presence of BC inflammatory emboli characterized by increased cell–cell adhesion with high E-cadherin expression and the hybrid E/M state [203,204]. Additionally, Chung et al. demonstrated that brain metastatic BCAF aggregates attract BC cells in vitro much more strongly than BCAF aggregates from the primary tumor (pBCAFs) or normal fibroblast aggregates. Brain metastatic BCAF aggregates express higher levels of CXCL12 (also known as SDF-1) and CXCL16 than normal breast fibroblast or pBCAF aggregates. Therefore, blocking the interaction between CXCR6-CXCL16 and/or CXCR4-CXCL12 significantly inhibits BC cell migration to brain metastatic BCAF aggregates. These data further support the important role of CXCL16 and CXCL12 in eliciting migration of CTCs to the brain metastatic niche [205].

Hemalatha et al. speculate that BC cells harboring mutations in genes involved in cell motility, such as BRCA1, may convert BCAFs into the so-called metastasis associated fibroblasts (MAFs), i.e., CAFs with enhanced capability to promote cancer cell migration, invasion and metastasis. They demonstrated that BCAFs treated with the conditioned medium of BRCA1-mutated BC cells acquire an altered phenotype, characterized by increased expression of metastatic and EpMT markers, including Ezrin, CCL5, and N-Cadherin. Interestingly, inhibition of Ezrin and CCL5 in MAFs reduces their capability to promote cancer cell migration and invasion in vitro. Therefore, Ezrin and CCL5 could represent promising targets to prevent the differentiation of BCAFs into a more metastatic phenotype, especially in BRCA1 mutated tumors [206]. A recent study demonstrated that pBCAFs and metastatic BCAFs (mBCAFs) isolated from skin, lung, liver, and bone metastases exhibit distinct gene expression profiles. In particular, mBCAFs show greater upregulation of genes related to the interferon (IFN) signaling pathway, ECM organization, hypoxia, and MAPK signaling pathway than pBCAFs do. The enhanced pro-tumorigenic and pro-metastatic properties of mBCAFs are partially due to increased secretion of IGF2. In fact, inhibition of the IGF signaling pathway not only dramatically decreases the mBCAF pro-tumorigenic phenotype [207] but also significantly inhibits lung metastasis in a TNBC xenograft model [208]. 

A recent study shows that the gene expression profile of fibroblasts co-evolves with the metastatic disease. In particular, each stage of the metastatic process can be defined by a specific gene signature that, in turn, correlates with a specific functional profile of fibroblasts. Therefore, the transcriptional rewiring of MAFs during the metastatic progression is fundamental for the generation of a permissive and suitable metastatic niche for cancer cell colonization and growth [209].

### 5.2. Role of BCAFs in BC Metastasis through ECM Remodeling

In some tumors, including BC, cancer cells start spreading long before the detection and the surgical removal of the primary tumor [210,211]. Interestingly, some tumors may be smaller, owing to the enhanced capability acquired by cancer cells to migrate away from the primary tumor mass and enter the circulation. This enhanced cell dissemination can be facilitated by the abnormal ECM found in tumors [210]. In this scenario, BCAFs regulate ECM composition and organization, and thus provide a favorable environment for BC dissemination and metastasis. Intriguingly, in vitro and in vivo studies show that BCAFs deposit an abundant and well aligned ECM, whereas normal fibroblasts from disease-free reduction mammoplasty tissues deposit an ECM with a random mesh-like organization. When non-malignant epithelial cells interact with BCAF-derived ECM, they acquire a mesenchymal phenotype, which is a necessary step for cell dissemination. In particular, these epithelial cells—which appear very elongated and regularly arranged in a parallel pattern—enhance the expression of the mesenchymal marker Thy-1, and upregulate the TGF-β-dependent Smad, Erk1/2, and Jun signaling pathways. Both non-malignant and malignant cells use the parallel organization of the ECM fiber bundles, generated by BCAFs, as tracks to disseminate in the blood circulation (Figure 3). Conversely, most of the time, normal fibroblasts not only fail to trigger the mesenchymal phenotype in epithelial cells but also suppress the tumorigenic and metastatic potential of malignant cells in vivo. Moreover, although the acquisition of the mesenchymal phenotype is necessary, it is insufficient to promote metastasis. Indeed, even when fibroblasts from disease-free reduction induce the mesenchymal phenotype in epithelial cells, they nonetheless fail to promote metastasis successfully. Therefore, only BCAFs can induce additional alterations in tumor cells, which consequently acquire a metastatic potential [210].

A major player in ECM remodeling is the tumor suppressor PTEN. PTEN downregulation and deletion characterize the activated stroma in solid tumors and about 50% of invasive BC, respectively [212]. The loss of PTEN dramatically triggers stromal microenvironment remodeling, which is associated with increased collagen deposition around mammary ducts, MMP-9 expression and activity, and macrophage infiltration [212]. PTEN-null fibroblasts in normal bearing mice promote collagen alignment parallel to that of the mammary duct [212]. This collagen organization resembles that of the so-called tumor-associated collagen signature 2 (TACS-2), which is characterized by a parallel linearization and organization of collagen at the tumor edge [212,213]. These results suggest that loss of PTEN in fibroblasts may prime gland mammary microenvironment for tumor development [212]. In addition, PTEN-null fibroblasts in tumor bearing mice increase the number of fibers oriented perpendicularly to the tumor edge. This perpendicular collagen orientation closely resembles that of the TACS-3, which creates tracks for cancer cells to spread beyond the primary tumor [212,213]. This is consistent with in vitro studies showing that murine mammary adenocarcinoma cells cultured on the ECM derived from PTEN-null fibroblasts are characterized by enhanced contractility and migratory capability [212]. Interestingly, inhibition of fibroblast contractility abolishes the increased collagen alignment normally observed with PTEN loss. Therefore, the signaling pathways involved in fibroblast contractility, such as the Rho/ROCK pathway, may represent potential targets to reduce BC metastasis caused by CAF-mediated ECM remodeling [214]. For instance, the ROCK inhibitor fasudil, approved by the Food and Drug Administration for the treatment of cerebral vasospasms, dramatically reduces BC metastasis in a BC mouse model [215]. Furthermore, it has been reported that TACS-3 is associated with significantly decreased overall survival in BC patients [216], suggesting that changes in collagen organization observed with loss of stromal PTEN may evolve with tumor progression [212]. 

Another player in ECM remodeling is the IGF/IGF-R1 signaling pathway. Indeed, BC cells, via IGF secretion, activate the IGF/IGF-R1signaling pathways in CAFs. In turn, the IGF/IGF-R1 axis promotes fibroblast motility and upregulates the promigratory protease urokinase (uPA) and its receptor uPAR. The synergistic occurrence of these events drives ECM remodeling, thus enhancing BC cell migration and invasion [217]. Noticeably, ongoing clinical trials targeting the IGF/IGF-1R pathway in BC patients are exploiting anti-IGF-1R monoclonal antibodies (such as dalotuzumab, figitumumab, cixutumumab, ganitumab, R1507, AVE1642), anti-IGF-I and IGF-II monoclonal antibodies (MEDI-573 and BI 836845), and a small IGF-IR-tyrosine kinase inhibitor (OSI-906) as a strategy to slow down, and, in the best case scenario, to block metastatic spread [218].

Adding to the complexity of ECM remodeling and BC metastasis is the fact that distinct subtypes of BCAFs produce different types of ECM, which, in turn, can differently modulate BC progression and invasion. For instance, CD146^–^ BCAFs promote a higher number of metastases than do CD146+ BCAFs. Furthermore, clinical data have shown that CD146^–^ BCAFs in BC predict an increased risk of developing lymph node metastasis even in patients with small primary tumors (<5 cm). This enhanced capability to develop metastasis is partly due to the ability of CD146^–^ BCAFs to secrete an ECM enriched for pro-metastatic proteins. In particular, CD146^–^ BCAF-derived matrix contains numerous ECM regulators, such as LOX, and structural ECM proteins, such as FN1 and TNC, all of which are overexpressed in very aggressive tumors [219]. For example, when TNC activates the EGFR signaling pathway, it induces an invasive phenotype in BC cells. This may explain why TNC is often upregulated at the invasive tumor front of aggressive and metastatic BCs [145,220]. Conversely, the basement membrane proteins, which act as an important barrier to cancer cell invasion, are significantly decreased in CD146^–^ BCAF-derived matrix [219]. These results are consistent with other data showing a stronger overexpression of FN, biglycan, and LOX enzyme in BCAFs with higher metastasis capability [144,210].

In addition, compelling evidence has demonstrated that chronic inflammation plays a fundamental role in cancer development and progression. Tumor infiltrating immune cells, especially neutrophils and eosinophils, contribute to the generation and maintenance of the tumor inflammatory milieu by producing massively myeloperoxidase (MPO) and eosinophil peroxidase (EPO), respectively. Indeed, MPO and EPO are strongly upregulated in the stroma of human BCs [221] and in mouse model of breast tumors [222]. Interestingly, these inflammatory enzymes contribute to BC invasion and metastasis by modulating fibroblast behaviors. Particularly, MPO and EPO strongly promote human mammary fibroblast migration, as well as increasing the fibroblast production of collagen I and VI, MMP-1 and MMP-3, and COX-2 [223], all of which act as important drivers of BC invasion and metastasis [224,225,226,227,228]. Additionally, MPO and EPO promote ECM structural remodeling by inducing collagen fiber alignment, which significantly increases cancer cell adhesion and invasion [223]. 

MMPs have been identified as markers of poor prognosis in BC patients, since they modify the TME. In particular, MMPs drive cancer progression and metastasis by directly degrading the ECM to promote tumor cell migration, and by destroying the basement membrane of blood or lymphatic vessels to promote tumor cell intravasation and metastatic dissemination [229,230]. Several in vitro and in vivo studies have reported that CAFs induce BC cell migration, invasion, and metastasis by producing various MMPs, including MMP-1, -2, -3, -7, -9, -13, and -14 [223,227,230,231,232,233]. Despite the prior disappointing clinical trials implementing the use of broad spectrum, small molecule catalytic site inhibitors, the identification of the mechanisms, by which these specific MMPs drive BC development and progression, offers promising targets to prevent and/or reduce BC progression [229]. 

COX-2 is still another important inflammatory mediator involved in tumor angiogenesis, invasion, and metastasis [234]. It is massively produced by both the stromal and the cancer cell compartments [223,234]. High COX-2 levels are associated with worse prognosis in BC patients and can predict larger tumor size and multiple lymph node metastases [235]. Furthermore, COX-2 triggers CAF activation in the tumor mass, as well as shape ECM structure and function. Krishnamachary et al. demonstrated that COX-2 overexpression significantly increases the number of activated CAFs and the amount of collagen 1 fiber density and volume [234]. In the tumor mass, collagen 1 fibers are mainly synthesized by CAFs [234] and facilitate BC metastasis by promoting the ameboid movement of tumor cells [236]. These data are consistent with studies showing a correlation between high density of collagen 1 fibers and the risk of developing metastasis [237,238]. Conversely, COX-2 downregulation significantly decreases the number of CAFs in the primary tumor and favors the formation of fewer and smaller metastatic lung nodules [234]. It is notable that fibroblasts increase COX-2 expression in BC cells by inducing the transcriptional activity of the nuclear factor kappa-light-chain-enhancer of activated B cells (NF-κB). NF-κB-mediated transcription of COX-2 is also associated with enhanced MMP-14 expression and MMP-9 protease activity. 

In addition to these COX-2-driven molecular events that facilitate BC progression and invasion [239], COX-2-overexpressing BC cells can also promote indoleamine 2,3-dioxygenase (IDO) expression in fibroblasts. More specifically, IDO-expressing fibroblasts secrete large amounts of kynurenine, which induces E-cadherin ubiquitination and degradation, thus leading to BC cell migration [240]. Collectively, these data support the theory that disrupting the paracrine crosstalk between BC cells and CAFs constitutes a novel therapeutic strategy for preventing BC growth and metastatic dissemination. Moreover, COX-2 and IDO could represent promising targets for BC therapy. In fact, Chen et al. demonstrated that COX-2 and IDO inhibitors exert a strong anti-tumor effect on COX-2-overexpressing BCs. Moreover, they also demonstrated that simultaneous targeting of COX-2 and IDO markedly inhibits BC growth, despite not resulting in an additive effect [240]. Furthermore, the use of NSAIDs or selective COX-2 inhibitors has been intensively investigated for the treatment and prevention of several cancer types [241,242]. Using an animal model of BC, Harris et al. demonstrated that the use of celecoxib, a specific COX-2 inhibitor, decreases the incidence and size of BC significantly. These authors also showed that the commonly used NSAID ibuprofen induces potent anti-tumor effects in vivo, albeit to a lesser degree than celecoxib [243]. Consistently, several human epidemiologic investigations have confirmed the efficacy of NSAIDs not only in eliciting anti-tumor effects, but also in significantly decreasing the risk of BC [244,245,246]. In line with these findings, other compelling studies have reported that suppression of COX-2 expression in primary cutaneous myofibroblasts induces an in vitro anti-tumor phenotype [247,248].

Another crucial protein responsible for BC initiation, progression, and metastasis is Cav-1. Loss of Cav-1 in breast fibroblasts promotes BC progression and metastasis by generating the so-called “lethal” TME, which is associated with poor clinical outcome. In particular, Trimmer et al. demonstrated that Cav-1-deficient fibroblasts increase the production of proteins associated with collagen synthesis and processing, especially collagen VI [249], which is upregulated in BC patients prone to develop lymph node metastasis [250].

Taken together, these data suggest that the stromal collagenous ECM and BCAFs can represent promising targets for BC treatment. Maintaining a normal ECM environment and normalizing a matrix-rich desmoplastic stroma by targeting ECM proteins may offer plausible novel therapeutic strategies, able to block cancer growth and dissemination and enhance the delivery and efficacy of chemotherapy. For instance, Weaver et al., by reverting the malignant BC cell phenotype to the normal phenotype through ECM modification, demonstrated that the ECM is dominant in determining the cell phenotype over the cellular genotype. In particular, they showed that tumor phenotypes can be reverted by culturing BC cells onto a 3-D basement membrane in the presence of integrin β1 blocking antibodies. The “normalization” of the tumor cell phenotype in vitro is also maintained in vivo, where the malignant potential of tumor cells treated with β1-integrin blocking antibodies is reduced or totally lost [251]. Strikingly, pharmacological inhibition of αv integrin with GLPG0187 sharply reduces bone metastasis in BC mouse models [252]. A case in point is zoledronate. This drug, approved for the clinical treatment of patients with multiple myeloma and bone metastases [253], also inhibits BC growth, migration and invasion by targeting various ECM proteins, including integrins ανβ3, ανβ5, and α5β1, and Sdc-1, and by suppressing the expression of MMP-2 and MMP-9 [254]. Furthermore, targeting Sdc-1 with radioimmunotherapy has proved to be a successful therapeutic strategy for the treatment of mice with TNBC [255].

FN, highly expressed by BCAFs, represents another promising target for cancer therapy. Interestingly, the use of inhibitors of FN polymerization can be very beneficial for cancer therapy, since FN is not actively produced in healthy tissues, except for wounded tissues and during development. Therefore, the advantage of using FN inhibitors is that they have minimal off-target effects [256]. Hielscher et al. showed that the treatment of co-cultures of fibroblasts and BC cells with pUR4B, a small FN inhibitor, decreases not only the deposition and organization of FN in the ECM, but also the deposition of other ECM proteins. Furthermore, inhibition of FN also affects the vascular morphogenesis of endothelial cells cultured on a FN-null matrix. Overall, knowing that FN deposition participates in ECM assembly and supports angiogenesis is instrumental for the development of novel anti-tumor agents able to disrupt cancer and vascular cell interactions with an FN-rich matrix [257]. Indeed, Tabariès et al. demonstrated that the use of neutralizing antibodies targeting α5β1 or α2β1 complexes can efficiently inhibit the binding of claudin 2-overexpressing BC cells to FN and collagen IV, consequently reducing the ability of BC cells to metastasize to the liver [258]. Furthermore, Takai et al. demonstrated that pirfenidone (PFD), which is an anti-fibrotic agent and a TGF-β antagonist, inhibits tumor growth of TNBC in vitro by targeting CAFs and by significantly reducing the density of collagen 1 fibers. Conversely, in vivo PFD monotherapy inhibits tumor fibrosis and TGF-β signaling without affecting cancer growth and lung metastasis. Moreover, PFD in combination with doxorubicin synergistically thwarts BC growth and lung metastasis. On the other hand, doxorubicin alone has been shown to inhibit primary tumor growth, but not lung metastasis in in vivo experiments [259]. Losartan, a potent anti-fibrotic compound, inhibits collagen I synthesis by BCAFs both in vitro and in vivo. Interestingly, in vivo studies demonstrated that losartan enhances tumor penetration and distribution of drugs and, in particular, increases the anti-tumor effect of i.v. injected pegylated liposomal doxorubicin [260]. Notably, Hu et al. encapsulated losartan into hydrogels, in order to allow a sustainable release of the encapsulated drug after intra-tumoral injection. When they used it in combination with doxorubicin-loaded liposomes (Dox-L), the losartan-loaded hydrogels significantly improved the intra-tumoral accumulation and penetration of Dox-L. Remarkably, this combination therapy was able to delay primary tumor growth by 64% and lung metastasis by 80% compared to Dox-L monotherapy [261]. Therefore, losartan efficiently destroys the dense collagen network that tumors efficiently exploit to reduce the penetration and efficacy of chemotherapeutic drugs [260]. Another promising compound that inhibits BC tumor progression is the selective tyrosine kinase inhibitor imatinib mesylate. This compound has been shown to inhibit not only the proliferation and invasiveness of human epithelial BC cells, but also to act as a potent anti-fibrotic agent by inhibiting both fibroblast proliferation and fibroblast-derived collagen accumulation. In particular, imatinib mesylate exerts a potent anti-tumor effect in the stromal compartment by downregulating the synthesis of collagen I and III—the main collagen types in BCM [262].

Lastly, the implementation of nanomedicine in the fight against a variety of different tumors, not least BC, is gaining increasing momentum. For instance, it has been demonstrated that, by functionalizing the surface of nanoparticles, with LOX inhibiting antibodies, it is possible to suppress mammary cancer cell growth and invasion more effectively than soluble anti-LOX antibodies both in vitro and in vivo. These results, besides substantiating the theory that ECM normalization is a useful strategy for BC treatment, they also provide a proof of concept for treating cancer with higher efficacy and decreased side effects by using nanomedicine [263].

### 5.3. Role of BCAFs in BC Metastasis via Induction of Angiogenesis 

Angiogenesis and hypoxia, by supporting each other, play an essential role in the formation of BC metastasis [264,265]. In the rapidly growing tumor mass, the morphological and functional abnormalities of the vasculature often result in heterogeneously distributed hypoxic areas [266] whose low levels of oxygen, in turn, promote and sustain angiogenesis [267]. Reduction in the oxygen supply concerns and influences both cancer and stromal cell compartments [268]. Ren et al. demonstrated that hypoxia not only induces BCAF activation, but also indirectly provokes BCAFs to promote angiogenesis and cell motility via HIF-1α and its target gene G protein estrogen receptor (GPER). In particular, BCAF-derived GPER and HIF-1α cooperatively promote the transcription of VEGF and connective tissue growth factor (CTGF), which are involved in angiogenesis and BC invasion, respectively [266]. Furthermore, BCAFs promote angiogenesis by secreting high levels of SDF-1, a protein that mobilizes and recruits endothelial progenitor cells (EPCs) from the bone marrow into the tumor mass. In the tumor, EPCs differentiate into the tumor-associated vascular endothelial cells [269] lining tumor blood vessels, which represent escape routes for metastatic cancer cells. Additionally, BCAFs induce endothelial changes by targeting cells via soluble factors. In particular, BCAF-derived IL-1β enhances the expression of several adhesion molecules, especially Selp (Selectin P), and the vascular cell adhesion molecule 1 (VCAM1) in endothelial cells. These adhesion proteins facilitate intravasation and extravasation of cancer cells by increasing tumor cell adhesion and transmigration through the endothelium. In addition, BCAF-derived IL-1 β also sustains and enhances invasion of BC cells by upregulating MMP-1, -3 and -5 [270]. Notably, BCAF-derived SDF-1 enhances cancer cell migration by binding to the receptor CXCR4 expressed on cancer cells. Consequently, SDF-1/CXCR4 binding induces actin polymerization and pseudopodia formation in cancer cells. These events drive the metastatic spread of cancer cells beyond the borders of the primary tumor toward organs that constitutively express SDF-1, such as lungs, bones, lymph nodes, and the brain. This explains why SDF-1-rich organs represent the most common metastatic sites of BC [271]. Therefore, since SDF-1 is a key factor involved in the growth, angiogenesis, invasion, and metastasis of BC, pharmacologically targeting SDF-1 and its downstream signaling pathway could have a relevant therapeutic potential for BC. Indeed, Gründker et al. have demonstrated that inhibition of the SDF-1/CXCR4 signal axis by kisspeptin-10 markedly reduces BC cell invasion [272]. Remarkably, several in vitro and in vivo studies have indeed identified several peptides or small molecule inhibitors of CXCR4 able to inhibit BC growth and progression dramatically [273,274,275,276,277,278,279].

Recently, a very interesting study investigated whether podoplanin, a glycoprotein with a key role in lymphatic vasculature formation and function, could contribute to spurring the metastatic properties of BC cells. In particular, this protein is highly expressed by fibroblasts present in the peripheral stroma at the margin of BCs, and in close proximity to tumor blood vessels. As expected, the study indicated that podoplanin expression favors fibroblast migration in the tumor stroma. This finding explains why podoplanin-rich BCAFs are preferentially detected in the peripheral tumor zone. Furthermore, fibroblasts expressing high levels of podoplanin have been found to interact efficiently with endothelial cells, as well as affect the tumor vascular network by promoting pseudo-tube formation. In addition, the results of clinical studies showing the presence of a higher number of podoplanin-rich BCAFs in IDC than in ductal carcinoma in situ (DCIS), further corroborate the pivotal role of podoplanin-positive BCAFs in BC progression and dissemination [280]. Niemiec et al. hypothesized that podoplanin-positive BCAFs could travel with metastatic cancer cells from the primary BC toward lymph node metastasis. In fact, they found that whereas podoplanin-positive BCAFs were comparably present in podoplanin-positive primary BCs and corresponding lymph nodes, they were absent in the lymph node metastases deriving from podoplanin-negative primary BCs [281].

Furthermore, preclinical and clinical studies showing the involvement of Sdc-1 in promoting and enhancing BC angiogenesis have been equally revealing [142]. In particular, it has been hypothesized that Sdc-1, via its heparan sulfate (HS) chains, activates the proangiogenic factors FGF2 and VEGF, thereby extensively contributing to tumor angiogenesis [142,143]. This theory is also strongly supported by several other studies showing that HS chains require many growth factors to spark the tumor signaling pathways [282,283]. Consistently, heparanase, by enhancing MMP-9 expression, induces expression and shedding of Sdc-1 in myeloma and BC. Indeed, by cleaving the core protein of Sdc-1, which remains biologically active [284], MMP 9 promotes BC progression by stimulating both angiogenesis [142] and cell dissemination [144]. Therefore, targeting Sdc-1 and/or inhibiting MMP-9 or heparanase could represent yet another potential therapeutic strategy for BC [285].

## 6. Conclusions

In both normal and neoplastic mammary glands, the interactions of stromal cells with either normal epithelial cells or cancer cells represent principal regulators of mammary gland homeostasis and cancer development, respectively. During BC growth, BCAFs exploit many altered morphogenetic signaling pathways, which are instead tightly regulated during physiological mammary gland branching morphogenesis. The comparison of mammary fibroblast and BCAF interactions with normal and cancer epithelial cells, which affect mammary gland branching morphogenesis and tumor development, could facilitate the understanding of alterations driving BCAF differentiation, BCM remodeling and cancer dissemination. Furthermore, a better understanding of the mechanisms underlying the formation of the pre-metastatic niche by BCAFs can be of fundamental importance in BC research. It has been well established that fibroblast populations from early-stage IDC are represented by BCAFs deriving from the tumor zone, myofibroblasts from the interface zone, and normal fibroblasts from the non-tumorigenic zone [137]. Therefore, we believe that the analysis of the highly complex interactions between cancer cells and fibroblast populations before CAF differentiation offers an important opportunity to better understand the role of normal fibroblasts in solid tumor development and dissemination.
